# A Comparison of Gingival and Periodontal Health in Anterior Teeth Restored With Porcelain-Fused-to-Metal and Zirconia Crowns

**DOI:** 10.7759/cureus.85469

**Published:** 2025-06-06

**Authors:** Anuja Lugade, Srilakshmi J, Krishna Kumar Vaidya, Prathamesh Gaonkar

**Affiliations:** 1 Prosthodontics, Rajarajeswari Dental College and Hospital, Bangalore, IND; 2 Prosthodontics, AECS Maaruti Dental College and Hospital, Bangalore, IND

**Keywords:** gingival health, periodontal health, pfm crown, plaque accumulation, zirconia crown

## Abstract

Objectives: This study aimed to evaluate and compare the gingival and periodontal health of teeth restored with zirconia or porcelain-fused-to-metal (PFM) crowns in the maxillary anteriors.

Materials: This study was conducted in the Department of Prosthodontics, Rajarajeswari Dental College and Hospital, Bangalore, India. A sample size of 20 patients who require PFM or zirconia crowns was considered in this study, one group consisting of 10 patients rehabilitated with zirconia crowns and the other group consisting of 10 patients rehabilitated with PFM crowns. The respective patients are checked for the Plaque Index (PI) of teeth before tooth preparation using the World Health Organization (WHO) probe and gingival status using the Gingival Index (GI). The tooth preparation with a 2 mm subgingival finish line was done for the respective anterior teeth, followed by crown placement. The patient was followed up after one week for baseline reading, three months, and six months to evaluate the gingival and periodontal health of the restored teeth using the GI and PI. The readings showing the periodontal status were recorded for the anterior teeth before and after restoration with zirconia and PFM crowns.

Results: In summary, while both groups demonstrated stable oral hygiene scores over time, the zirconia group showed significantly better performance in maintaining lower scores for plaque and gingival health at the six-month mark, indicating a more favorable outcome for long-term oral hygiene.

Conclusion: Initially, no notable differences were observed in the scores immediately after cementation and at the three-month mark. However, at the six-month interval, the zirconia group exhibited significantly lower scores for both plaque and gingival health, indicating superior outcomes in maintaining oral hygiene compared to the PFM group.

## Introduction

The scope of restorative dentistry encompasses not only the restoration of tooth function and aesthetics but also the preservation and improvement of the health of surrounding periodontal tissues. Porcelain-fused-to-metal (PFM) crowns have been widely used due to their proven strength, durability, and satisfactory aesthetic results [1]. However, there is an increasing preference for metal-free restorations that offer enhanced esthetic qualities and improved biocompatibility, leading to a rise in the application of zirconia crowns, particularly in the aesthetically sensitive anterior maxillary region [2]. Zirconia, a ceramic material, is distinguished by its superior fracture resistance, chemical inertness, and a color and translucency that closely resemble natural teeth [3,4].

An essential consideration for the long-term success of crowns is their effect on the surrounding periodontal tissues. Crowns with subgingival margins can act as plaque-retentive sites, which may provoke gingival inflammation and subsequent periodontal issues [5]. The physical and chemical surface properties of restorative materials, such as surface roughness, play a critical role in bacterial adhesion and biofilm development. Generally, smoother surfaces discourage plaque accumulation and promote periodontal health [6]. Compared with metal-based crowns, zirconia restorations tend to exhibit lower plaque retention and improved compatibility with soft tissues, likely due to their favorable surface characteristics and biocompatibility [7,8].

Clinical studies investigating the periodontal response to zirconia versus metal restorations generally reveal similar or better outcomes with zirconia crowns [9,10]. For example, a systematic review by Sailer and colleagues (2015) found that zirconia prostheses integrate with soft tissues equally well or better than traditional metal-ceramic crowns [11]. Laboratory evidence further demonstrates that zirconia surfaces are less prone to harboring pathogenic biofilms compared to metal alloys [12]. In addition, clinical monitoring over periods of up to one year shows stable gingival conditions and minimal inflammation around zirconia restorations [13,14]. In addition to the restorative material, factors such as crown fit, emergence profile, and type of cement used can significantly influence periodontal health outcomes and must be considered when evaluating prosthetic success.

Despite these encouraging results, there remains limited data focusing specifically on the periodontal health outcomes of zirconia compared to PFM crowns in the anterior maxillary region, a zone where both esthetic and periodontal considerations are paramount [15]. This study aims to fill this gap by assessing and comparing gingival and periodontal health in maxillary anterior teeth restored with zirconia and PFM crowns over a six-month follow-up, thus providing evidence to support clinical decision-making regarding material choice.

## Materials and methods

Study design

This comparative clinical study was conducted at the Department of Prosthodontics, Rajarajeswari Dental College and Hospital, Bangalore, India, over a period of 12 months on a total of 20 patients requiring anterior teeth restorations. This duration allowed for patient recruitment, treatment, and follow-up data collection at one week, three months, and six months after crown cementation. The participants were divided into two equal groups: Group A, comprising 10 patients rehabilitated with zirconia crowns, and Group B, consisting of 10 patients restored with PFM crowns fabricated using the conventional lost-wax technique followed by ceramic layering.

Sample size estimation

The sample size for the present study was estimated using the G*Power software (version 3.1.9.7; Heinrich-Heine-Universität Düsseldorf, Düsseldorf, Germany). The estimation was based on a priori analysis for a two-tailed test, with a 5% alpha error (α = 0.05), an effect size of 1.60 (as derived from a previous study by Pınar Kınay Taran and Mustafa Sarp Kaya (2018) evaluating differences in Gingival Index (GI) scores between the two groups during follow-up), and a study power of 80% (1-β = 0.80). The calculation indicated a minimum sample size of 16 samples (eight per group). To account for a potential 20% dropout rate during the follow-up period, the final sample size was increased to 20 patients, with 10 participants allocated to each group.

Patient selection

The inclusion criteria for patient selection were individuals aged 18 years and above requiring restorations for maxillary anterior teeth (canine to canine) with either zirconia or PFM crowns and presenting with clinically healthy gingiva and no debilitating systemic diseases. Patients were excluded if they exhibited poor oral hygiene, had any systemic condition compromising periodontal health, or presented with existing periodontal pathologies such as gingival recession, tooth mobility, or periodontal pockets.

Clinical procedure

**Figure 1:**
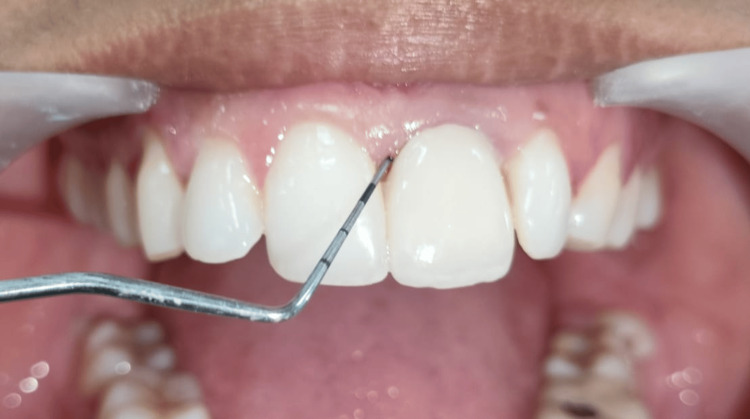
Evaluating the periodontal status using the WHO probe

Prior to tooth preparation, a baseline periodontal assessment was conducted by a single calibrated examiner. The clinical parameters recorded included the Plaque Index (PI) (Silness and Löe) and GI (Löe and Silness) using a WHO periodontal probe (Figure 1). A standardized tooth preparation protocol was employed for all patients by a single operator, involving a 2 mm subgingival finish line preparation on the selected maxillary anterior teeth. To assess the patients' perception of their oral health-related quality of life, the validated Oral Health Impact Profile (OHIP) questionnaire was administered. This self-reported questionnaire evaluates functional limitation, physical pain, psychological discomfort, physical disability, psychological disability, and social disability. The OHIP was completed by all participants at baseline and at the six-month follow-up to measure any changes related to the different crown materials.

In Group A, zirconia crowns were fabricated using the CEREC MC X milling machine with Upcera zirconia blanks. In Group B, PFM crowns were constructed using the conventional lost-wax technique, followed by ceramic layering. After crown fabrication, all restorations were cemented using Shofu HY-Bond GlasIonomer cement.

Follow-up and outcome assessment

Post-cementation, oral hygiene instructions were provided to all patients, emphasizing the Bass brushing technique. Periodontal evaluations were performed at three scheduled follow-up visits: one week (baseline after cementation), three months, and six months. During each follow-up, the PI and GI were recorded to monitor changes in periodontal health around the restored teeth. The primary outcome of the study was to assess and compare the periodontal status, specifically plaque accumulation and gingival health, of the anterior teeth restored with zirconia and PFM crowns over the designated follow-up period.

Statistical analysis

All statistical analyses were performed using IBM SPSS Statistics for Windows, version 22.0 (released 2013, IBM Corp., Armonk, NY). Descriptive analyses of all explanatory and outcome variables were conducted using means and standard deviations for quantitative data, and frequencies and percentages for categorical data.

The normality of data distribution was assessed using the Shapiro-Wilk test. Depending on data distribution, the independent Student's t-test (for normally distributed data) or the Mann-Whitney U test (for non-normally distributed data) was used to compare the mean Oral Hygiene Index Simplified (OHIs), PI, and GI scores between the two groups, and between treated and contralateral tooth sides at each follow-up interval.

To evaluate intra-group changes in OHIs, PI, and GI scores across different follow-up intervals, repeated-measures ANOVA (for normally distributed data) or the Friedman test (for non-normally distributed data) was applied. In cases where significant differences were detected, Wilcoxon signed-rank post-hoc tests were employed for pairwise comparisons. A p-value of <0.05 was considered statistically significant. Any additional statistical tests deemed appropriate during data analysis were applied accordingly.

## Results

Distribution of teeth

A total of 20 teeth were included in the study, with an equal distribution between the zirconia and PFM groups. In the zirconia group, 50.0% (n = 5) of the teeth were located in the upper right anterior region and 50.0% (n = 5) in the upper left anterior region. In the PFM group, 60.0% (n = 6) of the teeth were in the upper right anterior region, while 40.0% (n = 4) were in the upper left anterior region. Statistical analysis using the chi-square test revealed no significant difference in the distribution of teeth between the two groups (p = 0.65) (Table 1). This confirmed a balanced allocation of the tooth location, minimizing potential bias related to the anatomical site.

**Table 1:** Comparison of the distribution of teeth between the two groups using the Chi-square test PFM: porcelain fused to metal

Variable	Category	Zirconia		PFM		p-value
		n	%	n	%	
Teeth	Upper rt. anterior teeth	5	50.0%	6	60.0%	0.65
	Upper lt. anterior teeth	5	50.0%	4	40.0%	

Plaque Index (PI) scores

The intergroup comparison of the mean PI scores at different follow-up intervals is presented in Table 2. At one week following crown cementation (T1), the mean PI scores were 0.70 ± 0.68 in the zirconia group and 0.80 ± 0.63 in the PFM group. The difference was not statistically significant (p = 0.70). Similarly, at three months (T2), the mean PI scores were 0.80 ± 0.63 for zirconia and 0.90 ± 0.57 for PFM, with no significant difference (p = 0.69). However, at six months (T3), the zirconia group exhibited a significantly lower mean PI score of 0.60 ± 0.52 compared to 1.10 ± 0.32 in the PFM group (p = 0.02), indicating improved plaque control around zirconia crowns at this interval.

**Table 2:** Comparison of the mean Plaque Index (PI) scores between the two groups at different time intervals using Mann-Whitney test * The result is statistically significant.

Time	Crowns	N	Mean	SD	Median	Z	p-value
T1	Zirconia	10	0.70	0.68	1	-0.382	0.70
	PFM	10	0.80	0.63	1		
T2	Zirconia	10	0.80	0.63	1	-0.404	0.69
	PFM	10	0.90	0.57	1		
T3	Zirconia	10	0.60	0.52	1	-2.300	0.02*
	PFM	10	1.10	0.32	1		

Gingival Index (GI) scores

The comparison of the mean GI scores between the two groups at various time intervals is summarized in Table 3. At T1, the zirconia group recorded a mean GI score of 0.30 ± 0.48, while the PFM group had a mean score of 0.50 ± 0.53 (p = 0.37). At T2, the mean GI scores were 0.40 ± 0.52 in the zirconia group and 0.60 ± 0.70 in the PFM group (p = 0.55). Notably, at T3, the zirconia group demonstrated a significantly lower mean GI score of 0.20 ± 0.42 compared to 0.90 ± 0.32 in the PFM group (p = 0.002). This finding suggests a more favorable gingival response around zirconia crowns over a six-month period.

**Table 3:** Comparison of the mean Gingival Index (GI) scores between the two groups at different time intervals using the Mann-Whitney test

Time	Crowns	N	Mean	SD	Median	Z	p-value
T1	Zirconia	10	0.30	0.48	0	-0.890	0.37
	PFM	10	0.50	0.53	1		
T2	Zirconia	10	0.40	0.52	0	-0.602	0.55
	PFM	10	0.60	0.70	1		
T3	Zirconia	10	0.20	0.42	0	-3.067	0.002*
	PFM	10	0.90	0.32	1		

Intra-group changes in PI and GI scores

Intra-group changes in PI scores over the three follow-up intervals were assessed using Friedman’s test (Table 4). In the zirconia group, the mean PI scores were 0.70 ± 0.68, 0.80 ± 0.63, and 0.60 ± 0.52 at T1, T2, and T3, respectively, with no statistically significant difference over time (p = 0.61). Similarly, in the PFM group, the scores were 0.80 ± 0.63 at T1, 0.90 ± 0.57 at T2, and 1.10 ± 0.32 at T3, also showing no significant intra-group difference (p = 0.50).

**Table 4:** Comparison of the mean Plaque Index (PI) scores between different time intervals in each group using Friedman's test PFM: porcelain fused to metal

Group	Time	N	Mean	SD	Median	Min	Max	p-value
Zirconia	T1	10	0.70	0.68	1	0	2	0.61
	T2	10	0.80	0.63	1	0	2	
	T3	10	0.60	0.52	1	0	1	
PFM	T1	10	0.80	0.63	1	0	2	0.50
	T2	10	0.90	0.57	1	0	2	
	T3	10	1.10	0.32	1	1	2	

The intra-group evaluation of GI scores, as presented in Table 5, showed no statistically significant changes in the zirconia group across T1, T2, and T3 (p = 0.47). In the PFM group, the mean GI scores progressively increased from 0.50 ± 0.53 at T1 to 0.90 ± 0.32 at T3. Although the p-value approached significance (p = 0.07), it did not meet the conventional threshold, indicating a trend toward increased gingival inflammation over time in this group.

**Table 5:** Comparison of the mean Gingival Index (GI) scores between different time intervals in each group using Friedman's test PFM: porcelain fused to metal

Group	Time	N	Mean	SD	Median	Min	Max	p-value
Zirconia	T1	10	0.30	0.48	0	0	1	0.47
	T2	10	0.40	0.52	0	0	1	
	T3	10	0.20	0.42	0	0	1	
PFM	T1	10	0.50	0.53	1	0	1	0.07
	T2	10	0.60	0.70	1	0	2	
	T3	10	0.90	0.32	1	0	1	

## Discussion

The present study aimed to evaluate and compare the gingival and periodontal health of maxillary anterior teeth restored with either zirconia or PFM crowns over a six-month period. The results indicated that while both materials initially performed similarly in terms of plaque accumulation and gingival health, zirconia crowns exhibited a statistically significant advantage at the six-month follow-up.

These findings are consistent with earlier studies reporting that zirconia crowns tend to be more biocompatible and induce less inflammatory response in the surrounding gingival tissues compared to PFM crowns [16]. The superior performance of zirconia can be attributed to its highly polished, plaque-resistant surface and its minimal interaction with soft tissues, reducing bacterial colonization and subsequent gingival inflammation [17].

In the current study, no notable differences were observed immediately after cementation and at the three-month evaluation. This observation aligns with the understanding that early postoperative periods may not sufficiently reflect the long-term effects of restorative materials on periodontal tissues, as soft tissue adaptation and patient oral hygiene practices heavily influence early outcomes [18].

The significant differences noted at the six-month interval underscore the importance of material selection in long-term periodontal maintenance. Previous research has demonstrated that subgingival margins, especially when placed deep (2 mm subgingival in this study), can challenge plaque control and increase the risk of gingival inflammation if restorative margins are rough or biologically incompatible [19]. Zirconia’s favorable marginal adaptation and smooth finish could explain its better periodontal outcomes in this context.

Moreover, PFM crowns, while historically considered the gold standard, involve a metal substructure that can compromise gingival tissue health due to potential corrosion products and rougher marginal finishes, especially at the ceramic-metal junction [20]. This could contribute to increased plaque retention and a higher gingival index, as observed in the PFM group at the six-month follow-up.

In addition, studies have suggested that the optical properties of zirconia allow for better aesthetic integration without necessitating deep subgingival margins, indirectly supporting periodontal health by enabling more supragingival or equi-gingival placement when clinically feasible [21]. Although the present study standardized the margin placement at 2 mm subgingival for both groups, zirconia’s inherently favorable tissue response appears to have contributed to the lower plaque and gingival index scores observed.

The behavior of gingival tissues in response to different restorative materials has been extensively studied. Zirconia crowns tend to show favorable soft tissue compatibility and less inflammatory response compared to porcelain-fused-to-metal crowns [22]. Moreover, both materials have demonstrated comparable clinical survival rates, but the biocompatibility of zirconia may contribute to improved periodontal health outcomes [23].

A limitation of this study is the relatively small sample size and short follow-up period of 6 months. Future research with a larger cohort and longer observation periods would be valuable in corroborating these findings and assessing the long-term periodontal implications of zirconia versus PFM restorations. In addition, incorporating CAL (clinical attachment level) measurements and patient-reported outcomes regarding comfort and satisfaction could provide a more comprehensive evaluation.

In conclusion, this study reinforces the clinical advantages of zirconia crowns in maintaining better gingival and periodontal health over time compared to PFM crowns in anterior restorations. Given the increasing demand for highly aesthetic and biocompatible materials, zirconia appears to be a preferable choice for anterior restorations, particularly in patients where periodontal health is a critical concern.

## Conclusions

This clinical study demonstrated that while there were no significant differences in plaque and gingival health between zirconia and PFM crowns during the early postoperative period and at three months, a significant difference emerged at six months. Teeth restored with zirconia crowns exhibited significantly lower PI and GI scores compared to those restored with PFM crowns. These findings suggest that zirconia crowns may provide more favorable periodontal outcomes in anterior restorations over time. However, considering the limited sample size and follow-up duration, further studies with larger populations and longer observation periods are necessary to confirm these results and explore their long-term clinical implications.
